# Effects of Amprenavir on HIV-1 Maturation, Production and Infectivity Following Drug Withdrawal in Chronically-Infected Monocytes/Macrophages

**DOI:** 10.3390/v9100277

**Published:** 2017-09-28

**Authors:** Ana Borrajo, Alessandro Ranazzi, Michela Pollicita, Rosalinda Bruno, Andrea Modesti, Claudia Alteri, Carlo Federico Perno, Valentina Svicher, Stefano Aquaro

**Affiliations:** 1 Department of Experimental Medicine and Surgery, University of Rome Tor Vergata, 00133 Roma, Italy; ana.borrajo@hotmail.com (A.B.); alessandro.ranazzi@uniroma2.it (A.R.); michela.pollicita@uniroma2.it (M.P.); claudia.alteri@uniroma2.it (C.A.); cf.perno@uniroma2.it (C.F.P.); valentina.svicher@uniroma2.it (V.S.); 2 Clinical Virology Group, Institute of Biomedical Research of A Coruña (INIBIC)-University Hospital of A Coruña (CHUAC), Sergas, University of A Coruña (UDC), 15001 A Coruña, Spain; 3 Department of Pharmacy, Health and Nutritional Sciences, University of Calabria, 87036 Rende, Italy; r.bruno@unical.it; 4 Department of Clinical Sciences and Translational Medicine University of Rome Tor Vergata, 00133 Roma, Italy; modesti@uniroma2.it

**Keywords:** amprenavir, Human immunodeficiency virus, monocytes/macrophages, protease inhibitors

## Abstract

A paucity of information is available on the activity of protease inhibitors (PI) in chronically-infected monocyte-derived macrophages (MDM) and on the kinetics of viral-rebound after PI removal in vitro. To fill this gap, the activity of different concentrations of amprenavir (AMP) was evaluated in chronically-infected MDM by measuring p24-production every day up to 12 days after drug administration and up to seven days after drug removal. Clinically-relevant concentrations of AMP (4 and 20 μM) drastically decreased p24 amount released from chronically-infected MDM from Day 2 up to Day 12 after drug administration. The kinetics of viral-rebound after AMP-removal (4 and 20 μM) showed that, despite an initial increase, p24-production over time never reached the level observed for untreated-MDM, suggesting a persistent intracellular drug activity. In line with this, after AMP-removal, human immunodeficiency virus 1 (HIV-1) infectivity and intracellular the p24/p55 ratio (reflecting virion-maturation) were remarkably lower than observed for untreated MDM. Overall, AMP shows high efficacy in blocking HIV-1 replication in chronically-infected MDM, persisting even after drug-removal. This highlights the role of protease inhibitors in preventing the establishment of this important HIV-1 reservoir, thus reducing viral-dissemination in different anatomical compartments.

## 1. Introduction

Human immunodeficiency virus 1 (HIV-1) establishes a chronic infection, which can lead to severe immunodeficiency despite treatment with potent combined antiretroviral therapy (cART). HIV-1 can evade immune response by several mechanisms, including the establishment of persistent infection within different cell types, including memory or naive T lymphocytes and monocyte-derived macrophages (MDM). In particular, monocyte-derived macrophages (MDM) represent an important HIV-1 cellular reservoir as they can survive to HIV-1 cytopathic effect for prolonged periods of time (particularly microglia or alveolar macrophages) [1,2,3,4,5,6,7,8]. Furthermore, MDM can disseminate in different anatomical compartments (including the brain), thus contributing to HIV-1 spread in the body of an infected patient [9,10,11].

The morphogenesis of HIV-1 in MDM is different from that observed in CD4+ T lymphocytes. Indeed, in MDM, HIV-1 can bud and accumulate within late endosomes [12,13,14,15]. These virion-laden vesicles are presumably transported to the cell surface, and the virus is released from the cell surface following cellular exocytosis pathways [13,14,16].

Among the different HIV-1 proteins, protease (PR) is fundamental for the proper maturation of newly-produced virions as it cleaves gag and gag-pol polyprotein precursors to generate the single gag and pol proteins [17,18,19,20,21], thus leading to the production of mature and infectious viral particles [22,23].

As a consequence of its activity, essential for virus infectivity, PR represents an important target for the currently available cART. HIV-protease inhibitors prevent cleavage of *gag* and *gag-pol* polyprotein precursors, thus determining the release of immature viral particles from infected cells [24]. In this light, protease inhibitors (PIs) acting at a post-integrational step are so far, the only drug-class capable of inhibiting HIV-1 replication in chronically-infected cells, including MDM.

So far, a paucity of information is available on the activity of protease inhibitors upon the release of mature gag proteins from HIV-1-infected MDM. In particular, the following clinically-relevant points have not been clearly elucidated yet: (i) is there a correlation between inhibition of the release of mature virus gag proteins and the overall infectivity of virus particles released from chronically-infected cells; (ii) the antiviral effect of protease inhibitors is sustained over time, even after drug removal.

Among the class of sulfonamide protease inhibitors, amprenavir (AMP) produces potent effect against HIV-1 replication in CD4+ lymphocytes, with demonstrated activity in HIV-infected patients.

To provide a specific answer to the two above questions, we evaluated the characteristics and the dynamic of HIV replication and infectivity in chronically-infected MDM treated with AMP in terms of viral replication and infectivity rebound and in terms of gag-pol maturation and the morphology of newly-produced virus, when treatment had been interrupted before.

## 2. Materials and Methods

### 2.1. Cells

Human primary MDM were prepared and purified as previously described [25,26,27,28]. Briefly, MDM were obtained from the blood of healthy HIV-seronegative donors. Peripheral blood mononuclear cells (PBMCs) were separated by Ficoll-Hypaque gradient centrifugation and seeded in plastic 48-well plates (Costar, Cambridge, MA, USA) at a density of 1.8 × 10^6^ cells/mL in RPMI 1640 (EuroClone, Milan, Italy) supplemented with 50 U/mL penicillin, 50 μg/mL streptomycin, 2 mM l-glutamine and 20% heat-inactivated, mycoplasma- and endotoxin-free fetal calf serum (Corning, NY, USA) (complete medium). Cell cultures were incubated in a humidified atmosphere with 5% CO_2_ at 37 °C. Non-adherent cells were removed 6 days after seeding by repeated gentle washing with warmed RPMI 1640, leaving a monolayer of adherent cells, which were incubated in complete medium as previously described [29,30,31]. Adherent cells obtained using this technique generally consisted of >95% differentiated MDM (at cytofluorimetric analysis, more than 95% of cells were CD14+, CD4+, CD3−). Experiments were led in at least 3 independent experiments. In each experiment, MDM derived from a single donor.

### 2.2. Anti-HIV-1 Drugs

In this study, AMP was used as the prototype of a drug acting at the post-integrational step and thus capable of inhibiting HIV-1 replication in the setting of a chronic infection. Stock solutions from pure AMP powder were dissolved in dimethyl sulfoxide (DMSO; Calbiochem, La Jolla, CA, USA) at a 20 μM solution and stored at −80 °C until used. In each experiment, the nucleoside reverse transcription inhibitor, zidovudine (AZT) was used as a prototype of a drug acting at a pre-integrational step, thus ineffective at inhibiting HIV-1 replication in chronically-infected MDM.

### 2.3 HIV-1 Isolates

A monocytotropic isolate of HIV-1, HIV-1_BaL_, was used in all experiments involving primary MDM. The characteristics and genomic sequence of this strain have been previously described [31,32]. The virus was expanded in human primary MDM, collected, filtered and stored at −80 °C before use [28]. HIV-1_BaL_ was used starting from a concentration of 35 ng (corresponding to 2.1 × 10^8^ genomes) and from a 5000 Tissue Culture Infectious Dose (TCID)_50/mL_ (as assessed by virus titration in MDM).

### 2.4. Assessment of Antiviral Drug Activity in Chronically-Infected MDM

MDM were defined as being chronically infected when no new rounds of infection occurred in cultures in vitro and the p24 production remained stable. For this purpose, MDM were challenged with 300 TCID_50/mL_ of HIV-1_BaL_ (in the absence of any drug) on Day 0. At the time-point in which chronic infection was established, MDM were carefully washed at least twice to remove any virus present in the supernatants, replenished with fresh complete medium and cultured under the same conditions as described before. HIV-1 p24 release in supernatants was assessed every 3 days starting from Day 7. On the basis of our previous experience [28,33,34], chronic infection is generally established 7–10 days after viral challenge (some variation being detectable among different donors). In the experiments described herein, drug treatment began 14 days after infection, when HIV-1 p24 production reached a plateau [28].

Fourteen days after viral challenge (herein after called Day 0), MDM were carefully washed at least twice to remove any virus present in the supernatants, replenished with fresh complete medium containing various concentrations of AMP or AZT and cultured under the same conditions as described before. Each drug concentration was run in triplicate or quadruplicate, while untreated controls were run in sextuplicate. Starting from Day 0, all MDM-containing wells were washed (every 2 days) and replenished with fresh medium containing the appropriate drug concentration, according to the experimental protocol.

In selected experiments devoted to assess the duration of AMP activity following its removal, the cells were extensively washed and the medium completely changed at Days 5 and 6 after the beginning of treatment. Half of the wells were subsequently cultured without drug while the other half were cultured in the presence of drug as before.

At the end of the experiment, MDM were harvested for electron microscopy (EM) and lysed for Western blot analysis (see below).

Virus production was assessed at each time point by analysis of HIV-1 p24 production with a commercially available Radio-Immuno-Assay (RIA) (DuPont, Wilmington, DE, USA).

### 2.5. Virus Infectivity

Infectivity of virus particles produced by HIV-1-infected MDM was evaluated on MDM obtained from a different seronegative donor exposed to serial dilution of supernatants from drug-treated or non-treated HIV-1-infected MDM. The infectivity was assessed after 21 days (Day 14 of drug treatment, Day 7 after drug removal) of infection by measuring the p24 production. The TCID_50_/mL was calculated according to the Reed and Muench method.

### 2.6. Ultrastructural Study

Cells for electron microscopy were fixed in 2.5% glutaraldehyde (Serva, Heidelberg, Germany, F.R.G.; No. 23114) in phosphate buffered saline (PBS) pH 7.4 at 4 °C. Subsequently, MDM were washed twice in PBS and then fixed in osmium tetroxide 1.33% for 2 h at 4 °C. After several washes in PBS, the cells were dehydrated in graded alcohol, transferred into toluene and embedded in Epon 812 resin, then allowed to polymerize in a dry oven at 60 °C for 24 h. Thin sections were examined on an Axioscope microscope (Zeiss, Jena, Germany). Ultra-thin sections were cut on a Richert microtome using a diamond knife, were stained with uranyl-acetate-lead-hydroxide and evaluated on a Philips electron microscope CM10 (Philips, Endhoven, The Netherlands).

Intracellular p24 levels’ determination: HIV-1 chronically-infected MDM were removed from incubation and washed twice with 2 mL of cold PBS, then the supernatants were removed and stored at −80 °C for intracellular p24 titration.

### 2.7. Western Blotting of Cell Cultures

Cells were lysed in ice cold Radio-Immunoprecipitation Assay (RIPA) buffer (50 mM Tris-HCl, pH 7.4; 250 mM NaCl; 50 mM NaF; 0.15% Triton X-100) containing a protease inhibitor cocktail (1 mM phenylmethylsulfonyl fluoride; 10 µg/mL pepstatin; 10 µg/mL leupeptin) and incubated for 20 min at 4 °C. Cell lysates were centrifuged at 13,000 rpm for 10 min at 4 °C. Protein concentrations were determined by the bicinchoninic acid (BCA) protein assay (Thermofisher, Monza, Italy). Cell lysates containing 10 µg protein mixed and equal volume of 2× Laemmli buffer were boiled for 5 min, separated by SDS-polyacrylamide gel electrophoresis (SDS-PAGE) and transferred to nitrocellulose membranes.

The membranes were blocked overnight with 5% bovine serum albumin (BSA) in tris buffered saline (TBS)-tween buffer and incubated overnight using the broadly-reactive mouse monoclonal antibody AG3.0, for 1 h at room temperature in TBS-tween buffer and 1% BSA, recognizing HIV-1 p24, p55 and p150 (gag-pol) (NIH AIDS Research, Rockville, MD, USA). Then, membranes were treated with the corresponding horseradish perroxidase (HRP)-conjugated secondary antibody, and immunoreactivity was detected with an Immun-Star HRP Chemiluminescent Kit (Amersham, Piscataway, NJ, USA). For the p24/p55 ratio quantification, the bands on the films were first scanned by the Epson software program, and then, the images were processed through the Scion Image analysis program (Houston, TX, USA) for the IBM PC based on the NIH Image on the Macintosh platform.

### 2.8. Statistics

Differences were considered statistically significant at *p ≤* 0.05. Statistical analyses were carried out with SigmaStat 3.0 (Jandel Scientific, San Rafael, CA, USA).

## 3. Results

### 3.1. Impact of Amprenavir in Inhibiting HIV-1 Replication in MDM

**Figure 1:**
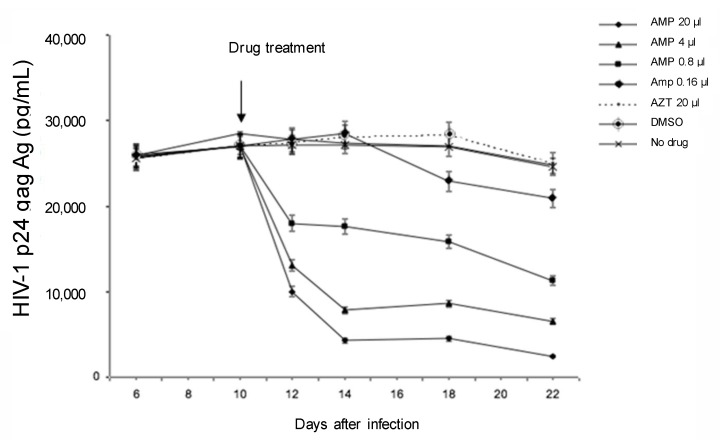
Kinetics of human immunodeficiency virus 1 (HIV-1) p24 gag Ag production in supernatants from HIV-1 chronically-infected monocyte-derived macrophages (MDM) treated with different doses of amprenavir (AMP). On Day 10 after infection, when HIV-1 infection becomes chronic, the drug was added to chronically-infected MDM. The figure reports data from a single experiment (each time point run at least in triplicate), which is representative of three different experiments.

We have previously demonstrated that AMP can substantially inhibit HIV-1 replication in acutely-infected MDM (treated before HIV-1 challenge) [29]. Thus, the first step of this study was to evaluate the antiviral activity of AMP in HIV-1 chronically-infected MDM. Viral replication in HIV-1-infected MDM treated with several doses of AMP was assessed by measuring p24 released in culture supernatant at 10 days after infection (time point at which there is a stable viral production). The highest concentrations of AMP (4 and 20 μM) determined a drastic decrease in p24 amount in the supernatant from Day 2 after drug treatment (corresponding to 12 days after infection) (Figure 1) Then, the p24 amount remained stable up to Day 12 after drug treatment (p24 decrease compared to untreated control: 73.2% for AMP 4 μM and 91% for AMP 20 μM). AZT at 20 μM was completely ineffective at blocking p24 release in chronically-infected MDM (Figure 1).

**Figure 2:**
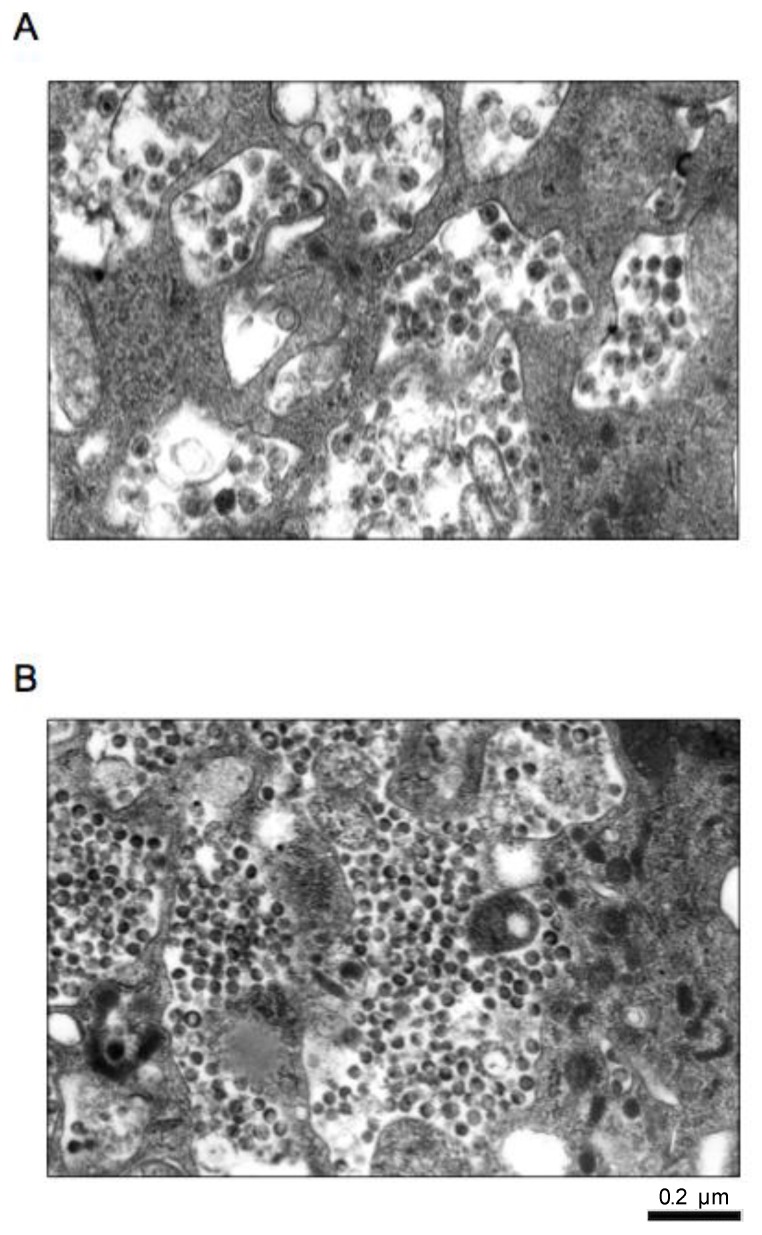
Photographs of electron microscopy representing: (**A**) viral particles contained in cytoplasmatic vacuoles in untreated HIV-1-infected macrophage; (**B**) immature virus particles contained in cytoplasmatic vacuoles in AMP-treated HIV-1-infected MDM.

By electron microscopy, in untreated HIV-1-infected MDM, viral particles had the typical electron dense conic-shaped core, indicating a proper maturation (Figure 2A). In addition, viral particles released in the extracellular compartment were more abundant than those present in cytoplasmic vacuoles (the latter a phenomenon typical only of HIV-infected MDM, but not CD4+ lymphocytes with a classical morphology). Conversely, in AMP-treated HIV-1-infected MDM, electron microscopy highlighted the presence of several immature viral particles inside cytoplasmatic vacuoles lacking the electron dense conic-shaped core, with altered organization of the envelope (Figure 2B).

### 3.2 Kinetics of Viral Rebound after Amprenavir Removal

**Figure 3:**
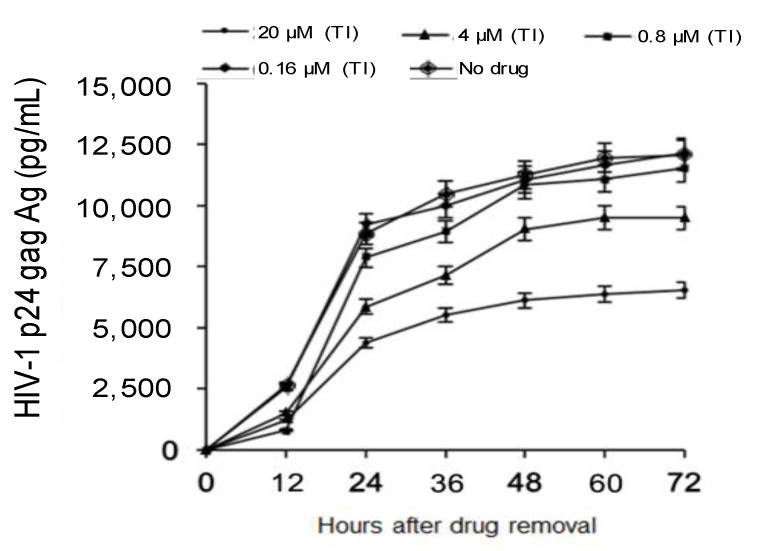
Kinetics of HIV-1 p24 rebound after AMP removal. HIV-1 p24 was measured in culture supernatants at 12, 24, 48 and 72 h after drug-removal from the supernatants of HIV-1 chronically-infected MDM. TI: treatment-interruption.

The second step of this study was to investigate the kinetics of viral rebound after AMP removal in HIV-1 chronically-infected MDM. To achieve this goal, p24 production in HIV-1-infected MDM was measured at different time points after drug removal. AMP was removed after seven days of treatment. At 12 h after drug removal, there was an increase in p24 production for all concentrations of AMP tested. As expected, for the lowest concentration of AMP (0.16 μM), p24 production over time reached levels observed for untreated MDM (Figure 3).

**Figure 4:**
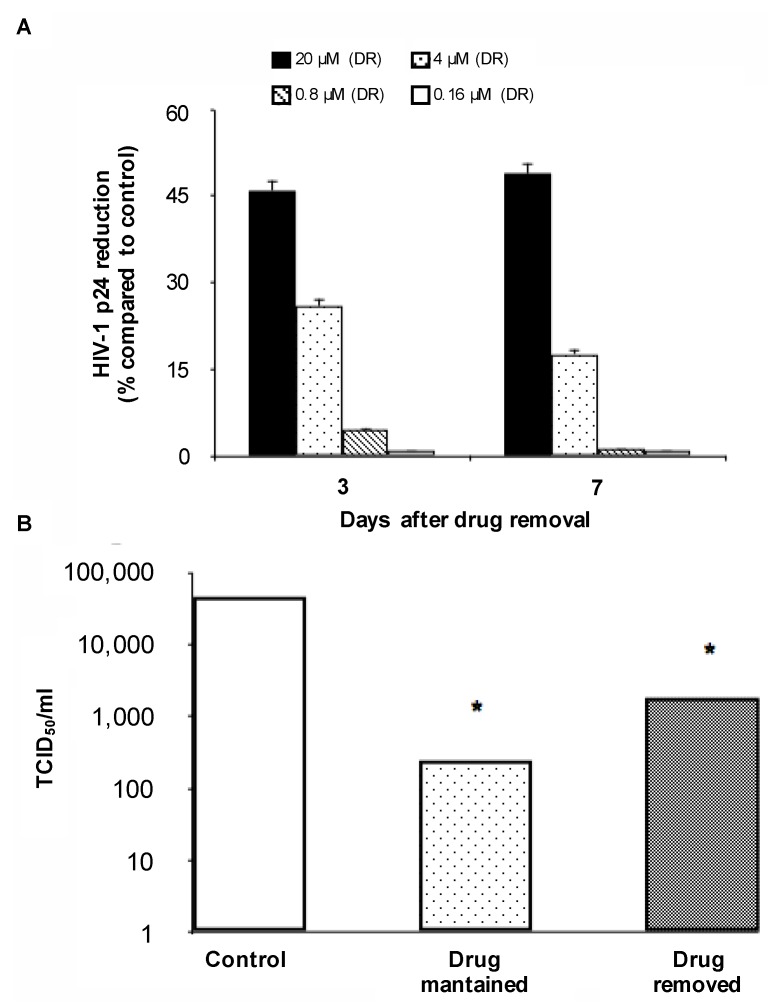
(**A**) Reduction of HIV-1 p24 production at three and seven days after drug removal in chronically-infected MDM. Statistical analysis was performed by the χ^2^ test. * *p* < 0.001; (**B**) HIV-1 titers (expressed as TCID_50/mL_) determined using supernatants from untreated HIV-1-infected MDM, AMP-treated HIV-1-infected MDM and HIV-1-infected MDM in which the drug was removed. Statistical analysis was performed by the χ^2^ test. * *p* < 0.001. DR: drug-removed.

A different behavior was observed for the highest concentrations of AMP. Indeed, after an initial increase, p24 production over time never reached the level observed for untreated MDM (Figure 3 and Figure 4). Notably, for AMP 20 μM, p24 production at three and seven days after drug removal showed a 46% and 49% reduction compared to untreated MDM, respectively (Figure 4A). Similarly, for AMP 4 μM p24 production at three and seven days after drug removal showed a 26% and 17% reduction compared to untreated MDM, respectively (Figure 4A). Overall, these results suggest a persistent intracellular activity despite drug removal from cell culture.

To confirm this hypothesis, the infectivity of viral particles released from infected MDM in which AMP was removed was compared with the infectivity of viral particles released from infected MDM in which AMP was maintained, as well as from untreated MDM. As expected, the highest titer was observed for untreated MDM. Interestingly, a significant decrease in TCID_50/mL_ was observed not only for drug-treated MDM, but also for those in which AMP was removed (Figure 4B).

**Figure 5:**
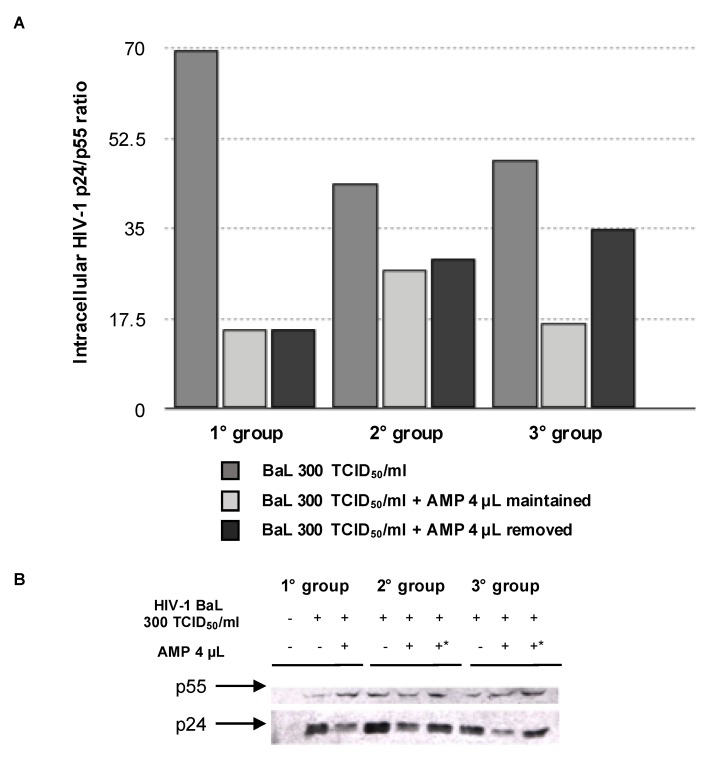
(**A**) Effects of AMP removal on p24 maturation in HIV-1 BaL chronically-infected MDM. p24 maturation was expressed as the ratio of p24 amount on p55 amount in cell lysates. p24 and p55 amounts were determined by ELISA; (**B**) western blot analysis showing p24 and p55 amount detected in cell lysates from chronically-infected macrophages. * Indicates the removal of AMP. In (**A**,**B**), the following experimental conditions were tested: Group 1, 18th day infection and 7th day treatment; Group 2, 18.5th day infection and 7.5th day treatment; Group 3, 19th day infection and 8th day treatment. HIV-1 BaL refers to an HIV-1 strain isolated from alveolar macrophages by a bronchoalveolar lavage.

In order to better explain the decreased HIV-1 infectivity, we evaluated p24 maturation in chronically-infected MDM after AMP removal. In particular, we measured the intracellular HIV-1 p24/p55 ratio in HIV-1-infected MDM at different days after drug removal. p55 is a p24 precursor; thus, the p24/p55 ratio can be considered a parameter to measure the maturation of the capsid. In all three experimental conditions, the p24/p55 ratio showed a drastic decrease, indicating an impairment in the maturation of the capsid despite the removal of the drug (Figure 5).

## 4. Discussion

This study shows that AMP is highly effective at inhibiting HIV-1 replication in chronically-infected MDM. Notably, this effect partially persists even after drug removal. These results are critical since MDM are an important cellular reservoir of HIV-1 infection, playing a critical role in HIV-1 transmission and dissemination throughout the body. There has been a recent resurgence of interest in the biology of monocyte subsets and MDM and their role in HIV-1 pathogenesis, partly fueled by efforts to understand difficulties in achieving HIV-1 eradication. Indeed, MDM are resistant to the cytopathic effect of the virus and thus accumulate replication-competent HIV-1 for prolonged periods, even in patients receiving cART [35,36,37]. Due to the pivotal role of MDM in the pathogenesis of HIV-1 infection, drugs able to inhibit HIV-1 replication in this cellular reservoir are needed. Only protease inhibitors showed the unique capability to affect the production of mature infectious virions from long-lived HIV-1-infected MDM.

In this study, we demonstrated that clinically-relevant concentrations of AMP (4 and 20 μM) efficiently affect HIV-1 production in HIV-1 chronically-infected MDM as attested by the substantial and stable inhibition of p24 release over time (up to Day 22 after treatment). We also found that a long-term anti-HIV-1 effect is partially maintained after drug removal with high concentrations of AMP in chronically-infected MDM, suggesting a persistent intracellular activity of the drug. Furthermore, a significant inhibition of infectivity was observed after drug removal, suggesting that the activity of the drug determines the production of non-functional viral particles. Again, these results confirm that AMP (and in general, protease inhibitors) may play a relevant role in inhibiting virus replication in chronically-infected MDM.

AMP inhibits the cleavage of the HIV-1 p55 gag precursor protein to the p24 and p17 core proteins [38,39,40,41], by interacting with high affinity to the substrate binding region of the mature viral PR dimer [27]. Thus, virions released in the presence of the protease inhibitor contained p55, little or no p24 and reduced RT. According to this, AMP (even after its removal) reduces the ratio of p24 on p55, indicating an impaired maturation of viral particles. This was further confirmed by electron microscopy, showing that AMP treatment determines the production of immature viral particles with the doughnut-shaped morphology in contrast to mature virions containing condensed cone-shaped cores observed in un-treated MDM. This is the first study addressing this issue in HIV-1-infected MDM, characterized by a mechanism of HIV-1 release different from that observed in CD4+ T lymphocytes. Again, the overall findings support the role AMP (and in general of this drug class) in controlling the establishment and enlargement of HIV-1 infection in this cellular reservoir. These findings are also important considering the results obtained in a recent study showing that immature virions produced in the presence of protease inhibitors are incapable of efficiently completing entry, reverse transcription and post-reverse transcription steps. This highlights the capability of protease inhibitors to block the HIV-1 life cycle at multiple steps [42,43]. These results support the high efficacy of protease inhibitors observed not only in cART, but also in simplification strategies. This is also clinically relevant in view of the pivotal role of MDM in contributing to establishing HIV-1 persistence in different anatomical compartments including the central nervous system.

Previous studies have shown that the pathway of HIV-1 particle morphogenesis can be different in primary human macrophages compared to CD4+ T lymphocytes. Indeed, it has been shown that HIV-1 can bud and accumulate within an intracellular vesicular compartment, mainly represented by late endosomes [9,10,12,44]. This intracellular HIV-1 accumulation has been proposed to play a pivotal role for pathogenesis and dissemination since HIV-1 can be retained in an infectious state for prolonged periods of time inside macrophages and may be released in a delayed manner similar to the secretion of exosomes. In this light, it can be hypothesized that AMP can be incorporated into viral particles within late endosomes, thus favoring a prolonged release of immature and non-infectious viral particles from chronically-infected MDM. The incorporation of AMP into viral particles within late endosomes may also hamper the metabolism of the drug by the cytochrome P450 isoforms expressed in monocytes/macrophages (CYP2E1 and CYP3A4), thus further contributing to persistent AMP activity.

Furthermore, a recent study has highlighted the contribution of tissue macrophages in establishing a persistent infection despite successful cART [45]. In this light, the potent and prolonged anti-HIV-1activity of amprenavir again supports the role of drugs acting at post-integrational steps in controlling HIV-1 replication in chronically-infected MDM.

Furthermore, recent studies have suggested HIV-1’s ability to establish a latent infection in macrophages [46,47]. In particular, a recent study demonstrated the presence of the latent macrophage reservoir in brains of SIV-infected ART-treated macaques [47]. The authors found that viral RNA in brain tissue of suppressed macaques was undetectable, although viral DNA was detected in all animals. In addition, they found that virus produced in latent macrophages is replication competent, suggesting that latently-infected macrophages are capable of re-establishing productive infection upon treatment interruption. The overall findings support the existence of latent reservoirs other than resting CD4+ T lymphocytes and underscore the importance of macrophages in developing strategies to eradicate HIV [47]. In this light, the use of drugs acting at the post-integrational step appears critical in order to prevent HIV-1 reactivation in latently-infected macrophages. This is critical also considering a previous study showing that resting CD4+ T cells isolated from PI-treated patients compared to non-nucleoside reverse transcriptase inhibitor (NNRTI)-treated patients showed a limited HIV-1 reactivation upon T-cell stimulation [46]. This suggests that PI-based cART could be more efficient than NNRTI-based cART in limiting HIV-1 reactivation in aviremic chronically-infected patients.

In conclusion, AMP shows high efficacy in blocking HIV-1 replication in MDM. This highlights the role of this drug (and in general of protease inhibitors) in controlling the establishment and enlargement of this important HIV-1 reservoir. Due to the role of MDM in disseminating HIV-1 in different body compartments, this is also critical to efficiently hit HIV-1 replication in all anatomical compartments where the virus hides and replicates.
